# Examining the Effects of One- and Three-Dimensional Spatial Filtering Analyses in Magnetoencephalography

**DOI:** 10.1371/journal.pone.0022251

**Published:** 2011-08-03

**Authors:** Sam Johnson, Garreth Prendergast, Mark Hymers, Gary Green

**Affiliations:** 1 York NeuroImaging Centre, University of York, York, United Kingdom; 2 Hull York Medical School, University of York, York, United Kingdom; Beijing Normal University, China

## Abstract

Spatial filtering, or beamforming, is a commonly used data-driven analysis technique in the field of Magnetoencephalography (MEG). Although routinely referred to as a single technique, beamforming in fact encompasses several different methods, both with regard to defining the spatial filters used to reconstruct source-space time series and in terms of the analysis of these time series. This paper evaluates two alternative methods of spatial filter construction and application. It demonstrates how encoding different requirements into the design of these filters has an effect on the results obtained. The analyses presented demonstrate the potential value of implementations which examine the timeseries projections in multiple orientations at a single location by showing that beamforming can reconstruct predominantly radial sources in the case of a multiple-spheres forward model. The accuracy of source reconstruction appears to be more related to depth than source orientation. Furthermore, it is shown that using three 1-dimensional spatial filters can result in inaccurate source-space time series reconstruction. The paper concludes with brief recommendations regarding reporting beamforming methodologies in order to help remove ambiguity about the specifics of the techniques which have been used.

## Introduction

Magnetoencephalography (MEG) is a non invasive neuroimaging technique now commonly used to investigate human neural processes [1]. The technique measures the magnetic fields outside the head produced by neuronal activity. As such there is a need for source modelling techniques to project the measured sensor data into the source space of the brain. One of the principle techniques used is a spatial filtering technique referred to as beamforming [2], [3]. Beamforming is a data-driven scanning technique which requires no a-priori modelling of the number of underlying sources, and can, in theory, evaluate the signal at all locations within the brain.

The underlying principle of beamforming is that, given a set of spatially distributed sensors and data over time, it is possible to calculate a set of weights such that a source at a particular location within the brain can be reconstructed using these weights to linearly combine the sensor-space data. This reconstructed source is often referred to as a virtual electrode (VE). This VE is then characterised by a metric which quantifies some aspect of the VE time series thought to be of interest. The most commonly used metric in beamforming is based upon power, the Neural Activity Index (NAI) or pseudo-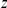 statistic. These metrics are then commonly compared using a true- or pseudo-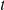 statistic in order to look for differences between periods of active and passive data [4], [5]. Although power is the most commonly used method of assessing beamformer outputs, other metrics such as event-related measures [6], [7], inter-trial coherence [8] and correlation-based measures (e.g. [9], [10]) have been used. In addition, the spatial filtering approach has been used in the frequency domain to look at coherence across brain regions [11], [12].

There are various approaches to the calculation of the spatial filters, all of which derive from the formalism described by Van Veen et al [4]. Huang et al [13] provide a comparison of several different beamformer implementations and it is noted that they primarily differ in their application of the noise normalisation process. Noise normalisation is necessary due to inhomogeneities in signal-to-noise ratio throughout the volume. Estimates of power are therefore biased by differing levels of noise making comparisons problematic. Normalisation is performed in an attempt to estimate purely signal-based power, thus allowing more accurate comparisons to be made. As well as changing throughout the volume, signal-to-noise ratios will also be different at different orientations at a specific location. Huang et al refer to the Van Veen approach as a “Type-I” beamformer and make the point that the noise normalisation described in equation 27 of [4] (repeated in this paper as equation 8) may result in orientations with poor signal-to-noise ratios dominating real signals coming from other orientations. To address this issue Huang et al propose a modification of this normalisation in which each orientation is normalised with respect to its own noise. The implementation of this modification however, has the effect of altering the calculation of the spatial filter. This issue is the main focus of this paper and the details will be discussed in the methods section.

Another major difference between beamformer implementations is whether they are considered scalar (non-linear) or vectorised (linear) beamformers. The difference between the two is in whether any given metric, for example pseudo-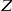, is assessed in a single orientation (scalar) or multiple orientations (vector) for each location within the volume. There are a variety of methods of choosing which orientation is used in a scalar beamformer implementation. Some scalar beamformers also restrict analysis to the tangential plane (for example, Synthetic Aperture Magnetometry or SAM [14]) although this is not inherently required by the technique. In this paper, we will use the terminology of scalar-output and vectorised-output beamformers, to refer to either a single or multiple source reconstructions for a given location.

An often discussed issue in MEG analysis is the problem of radial sources. In the limit an isolated neural current which is completely radial to a magnetometer or gradiometer will result in no signal being measured. Although modern MEG systems typically have well in excess of 200 channels, all of which are oriented differently, it is considered that radial sources will be difficult to detect using MEG. This is partially due to the traditional constraints of the single-sphere forward model in which radial sources in the model will be invisible [15]. Despite the fact that in multiple-spheres forward models [16], no direction will be considered truly radial to all sensors, various applications restrict themselves to the tangential plane.

Whilst using a scalar-output beamformer and restricting to the tangential plane reduces the dimensionality of the data and avoids the previously discussed issues with predominantly radial sources, is it possible that information may be lost by adding these constraints. Hillebrand and Barnes [17] provide evidence for this in their investigation of the sensitivity of MEG using gradiometer-based sensors and equivalent current dipoles, in which they concluded that the depth of a source was considerably more important in affecting its detectability than the orientation of that source. It is therefore important to examine how well the beamformer approach can reconstruct known signals in multiple orientations.

In this paper, we will study the accuracy of VE reconstructions throughout the brain when using a vectorised beamformer to recover synthetic, known, sources. We will also compare different methods of constructing a spatial filter. Although the differences between spatial filter construction methods are present in the initial literature, many papers do not make it clear which filter construction method is in use for any given study. The advantages and drawbacks of each method will be described as well as the importance of ensuring clarity in how the spatial filter was calculated when reporting beamformer findings.

## Results

The mathematical notation used in this paper is that a lower case, standard font letter, 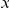, is a scalar, a lower case, bold font letter, 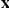, is a vector and an uppercase, bold font letter, 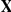, is a matrix. Details of the terminology used in this paper can be found in table 1.

**Table 1:** Definitions of mathematical terms used in the text.

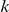	Spatial location
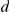	Spatial orientation
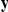 , 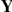	Virtual-Electrode time series reconstruction
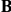	MEG sensor data
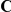	Estimate of MEG sensor data co-variance ( 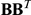 )
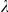	Smallest eigenvalue of 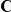
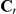	Regularised estimate of MEG sensor data co-variance ( 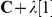 )
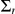	Regularised estimate of MEG sensor data noise c0-variance
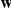 , 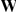	Spatial-filter defining weights (a number of sensors by 1 vector or number of sensors by 3 matrix respectively)
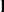 , 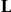	Leadfield (a number of sensors by 1 vector or number of sensors by 3 matrix respectively)
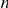	Neural Activity Index
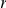	Radial direction, defined using average sphere centre
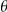 , 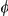	Two orthogonal directions in the tangential plane

### Spatial filters

There are in fact two distinct interpretations of a “scalar” beamformer, i.e. scalar in the output and scalar in the filter. Similarly a “vector” beamformer can be a vectorised output of a beamformer or a vectorised, 3-dimensional filter implementation. Throughout this paper, we will use the conventional definitions of “scalar” and “vector” to describe the nature of the output of the filters. When discussing the generation of the filters, we will describe them as either “three 1-d filters” or “one 3-d filter”.

The work described in this paper uses a vectorised, linearly constrained minimum variance beamformer as described by Van Veen et al [4] and referenced in Huang et al [13] as a “Type I” beamformer. Although these implementations are superficially similar, there is an important difference in the calculation of the spatial filter. Van Veen et al showed how a spatial filter could be constructed that will minimise the power transmitted through the filter whilst maintaining unit gain at a position and orientation of interest. This will, in general, minimise the power from all sources not at the position and orientation of interest.

A more detailed derivation can be found in Van Veen et al [4], but the ideal spatial filter is defined such that its inner product with the measured magnetic field gives the source at the location of the spatial filter, i.e.:
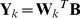
(1)This is equation 11 in Van Veen et al [4], extended for multiple time points. Huang et al [13] describes similar filters in his equation 4, but instead defines single orientation filters:
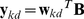
(2)where now 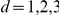, which are three orthogonal directions.

Equations 1 and 2 are equivalent. Equation 1 is simply a shorthand way of expressing the three instances of equation 2. If the weights in equation 1 are a concatenation of the weights in equation 2 then the the output of equation 1 will also be a concatenation of the output of equation 2, i.e. if:
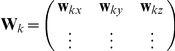
then,
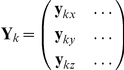
In this ideal situation the spatial filter can also be defined in terms of its pass characteristics, that is the gain of the filter for different locations and orientations. This is simply a product of the weights of the spatial filter and the leadfield at a given location and orientation, 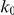.
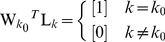
(3)where the leadfield, 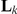 is generated for every grid point in the head and describes the sensitivity profile of each of the sensors in the array to that position. For the work carried out in this paper the leadfields were calculated using a multiple spheres model [16].

### Calculating the weights

In reality of course, such an ideal spatial filter cannot be constructed. Instead the spatial filter is optimised by minimising the total power it passes, with the constraint of unity gain for the point and orientation of interest. This is performed using a Lagrange multiplier, the exact form of which marks the difference between the Van Veen and the Huang Type I beamformer.

In Van Veen the power is given by:
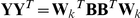


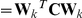
and is minimised subject to the ideal constraint of equation 3 applied only for the position of interest, i.e.:
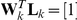
This treats the reconstruction of the sources as an integrated, 3-dimensional problem and therefore ensures that the gain at a given position is unity in the orientation of interest and zero for orthogonal orientations:
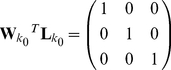
(4)In Huang however, the power is given by:
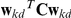
and is minimised subject to:
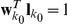
This apparently minor alteration is in fact a relaxing of the constraint and only ensures that the gain at a given location in the orientation of interest is unity. This treats the reconstruction as three independent 1-dimensional problems. This makes the Huang equivalent of equation 4:
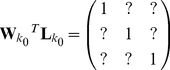
(5)where now the 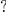
s in the off diagonal positions are left undefined.

In both cases the spatial filter is constructed using weights, 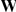 or 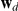, generated for every grid point 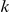. These weights are calculated using an estimate of the covariance of the data 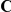 and the leadfield 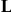 or 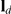 as described by equation 6 or 7.
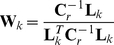
(6)

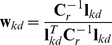
(7)Here 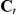 is the regularised version of the estimate of the covariance. For the work carried out in this paper regularisation was applied to the calculation of the covariance matrix using the smallest eigenvalue of 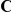. The regularised covariance matrix is therefore 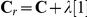. In general 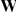 will not be a concatenation of 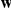.

### Source reconstruction

The equation for calculating the neural time course at a given location in a given orientation (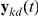) is the same across beamformers in that it is the inner product of the weights and the MEG sensor data. This reconstruction is given by equation 1 when treated as an integrated three dimensional problem (where 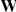 is given by equation 6 above), and by equation 2 when treated as three separate, one-dimensional problems (where 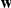 is given by equation 7 above).

For the analyses presented, both beamformers were implemented as vectorised output beamformers, and therefore time series reconstructions were generated in three orthogonal directions, either as the three separate outputs of three 1-d filters or the three dimensional output of one 3-d filter. To investigate the sensitivity of MEG to predominantly radial sources, the co-ordinate system used was a point specific one, with 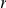, 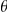 and 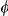 unit vectors defined at each point. As the leadfields were implemented using a multiple spheres model there is no no truly radial direction at a given location. An approximately radial unit vector, 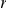, was defined using the mean of the sphere centres as the origin. Two orthogonal unit vectors 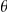 and 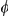 were then defined in the tangential plane.

### Localisation and Normalisation

As well as the accuracy of source reconstructions, it is important to consider the related but separate issue of the accuracy of source localisations. As previously noted, source localisation is complicated by the inhomogeneous distribution of signal to noise throughout the brain volume. When considering the reconstructed time course of a phase locked source, this effect is ameliorated by the averaging process and the assumption that any noise components will not be phase locked. Both the Van Veen and Huang Type-I beamformers normalise the source power by 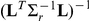, but again the difference is whether the system is treated as one 3 or three 1 dimensional problems. Van Veen describes his NAI in his equation 27 as:
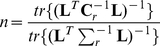
(8)Huang expands this in his equation 10 as:
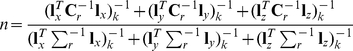
(9)however this only holds if the 3×3 matrices 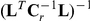 and 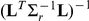 have zero off-diagonal terms. This is analogous to equation 5, where the Huang formalism ignores the off-diagonal terms. In fact it is the off-diagonal terms that contain the information that allows the constraint of equation 4 to be met. Huang does make the point though, that in either way of describing the NAI there is the possibility that a noisy orientation, i.e. with a small leadfield, will dominate the NAI. Huang proposed a modification that avoids this problem by normalising in each orientation separately:
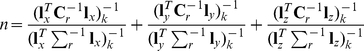
(10)Although in the original papers the co-ordinate system used was a cartesian 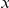, 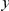, 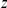 system, these equations hold for any orthogonal 3-space co-ordinate system including the 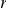, 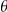, 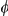 system used here to systematically investigate the reconstruction of predominantly radial sources.

In the following results, the Van Veen implementation of a beamformer (equation 6) will be described as a one 3-d spatial filter and the Huang implementation (equation 7) will be described as three 1-d spatial filters.

### Reconstruction in the correct orientation

**Figure 1:**
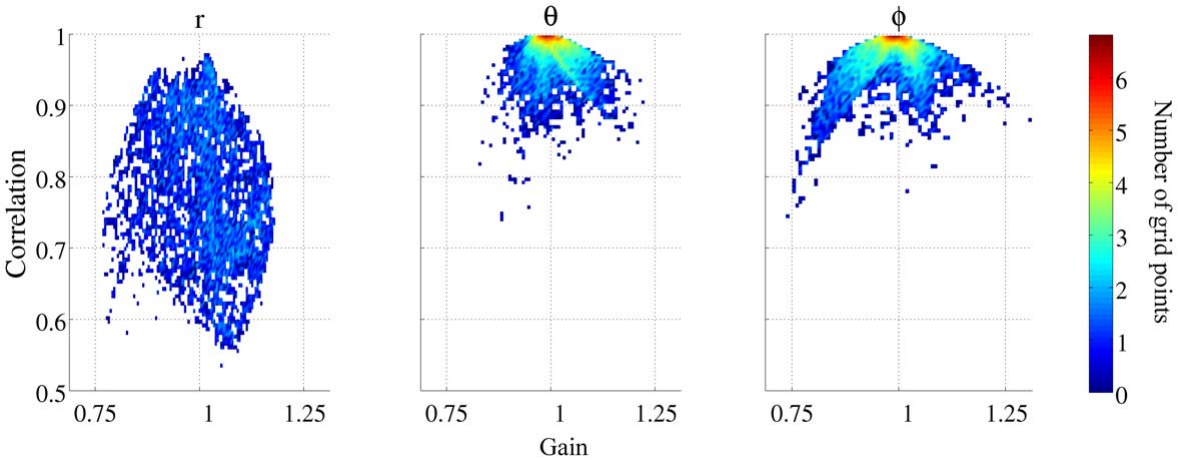
Two dimensional histogram of gain and correlation of the reconstruction compared with the embedded signal at each location in the brain volume for three 1-dimensional filters. The left, middle and right columns show the results for sources both embedded and reconstructed in the 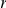, 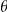 and 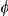 directions respectively.

**Figure 2:**
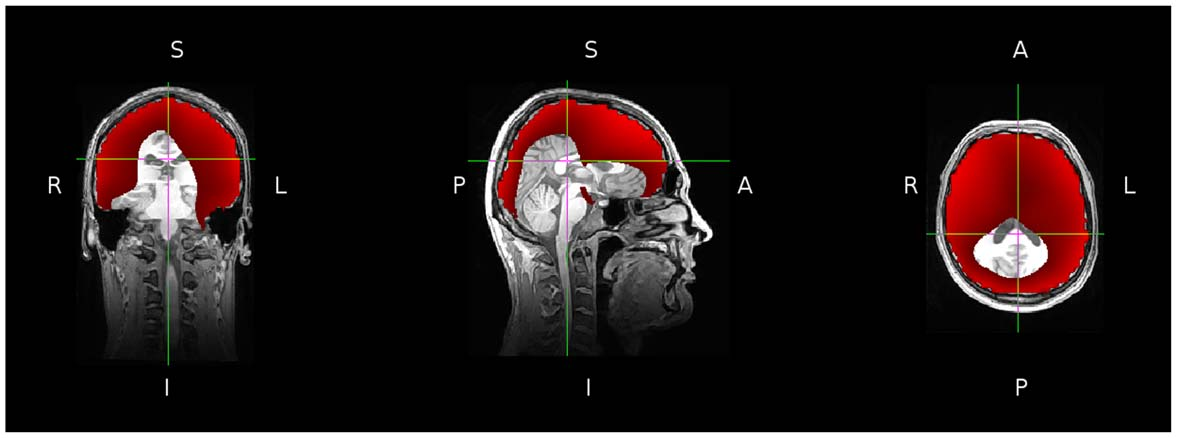
Volumetric image showing correlations between sources embedded in the 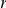 direction and the reconstructed signals when using three 1-dimensional filters. The image is thresholded at 0.7.

Figure 1 shows a two-dimensional histogram for the gain and correlation of the source reconstruction obtained using three 1-d spatial filters at each grid location. The left, middle and right-hand panels show the reconstructions in the 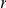, 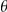 and 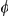 directions respectively. Inspection of the figure shows that sources seeded in a predominantly radial orientation are not reconstructed as accurately as in the two non-radial directions. The mean correlation in the tangential directions was 0.96 (std = 0.06) whereas it was 0.77 (std = 0.10) for the predominantly radial direction. The highest correlation in the 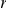 direction was 0.98, whilst 72.40% of grid locations showed a correlation greater than 0.7. (The figure of 0.7 allows comparison with Hillebrand and Barnes [17].) This percentage increases to 99.32% and 99.14% in each of the two non-radial directions. No grid locations had a reconstruction with a gain magnitude greatly displaced from 1 (range −0.86 to 1.15). Figure 1 suggests that in a non-radial direction, a three 1-d spatial filter implementation of a vectorised beamformer is able to reconstruct a known source with a high correlation and an accurate gain. Although the correlations observed in the 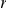 direction are not as high, it is inaccurate to describe this beamformer as unable to reconstruct predominantly radially oriented sources. Figure 2 shows a volumetric map of correlations seen for each point when a source was seeded in the 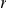 direction. The image has been thresholded at 0.7, and it is clear that the volumetric locations with low correlations are found at deeper rather than superficial locations.

### Reconstruction in the off orientations

The results shown in figure 1 provide an estimate of how effective a tool a vectorised beamformer might be and confirm that such an approach is able to accurately reconstruct signals throughout the volume and also in predominantly radial directions. These results, however, were obtained by reconstructing the time series in the same orientation as the signal was placed, i.e. in a known orientation. In contrast, in a typical analysis using an observed response evoked by some stimulus, the orientation of the sources will be unknown. It is therefore important to examine signal reconstructions in the off-directions when using three 1-d spatial filters.

**Figure 3:**
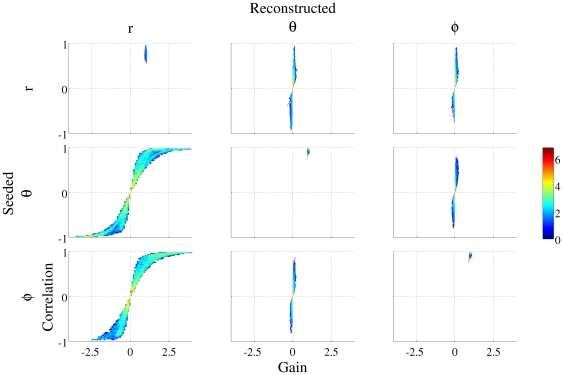
Two dimensional histograms of gain and correlation of the reconstruction compared with the embedded signal at each location in the brain volume for three 1-d filters. The upper, middle and lower rows represent sources *seeded* in 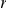, 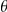 and 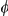 respectively; whilst the left, middle and right hand columns show *reconstructions* in 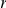, 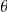 and 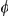. The diagonal of these figures shows the same data as that in Figure 1.

Figure 3 shows the gain and correlation information for sources *seeded* in 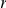, 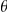 and 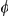 (upper, middle and lower rows respectively) whilst the columns represent the *reconstructions* in each of these orientations. The information on the diagonal of this figure is therefore the same information as is shown in figure 1.

In the off-directions, ideally there would be a gain and a correlation of zero due to the fact that no source was seeded in that specific orientation and location.

The reconstructions in the two non-radial directions (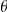 and 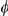) when no source was placed in this orientation, show predominantly small gains (81.48% of all reconstructions had a gain of zero +/−0.1). However, the correlation of the reconstructed waveform and the seeded waveform can be high, despite the fact that the embedded signal was placed in a different orientation (5.03% of the volume showed a correlation stronger than 0.7). Therefore the signal placed in one orientation is “leaking” through into the other directions.

**Figure 4:**
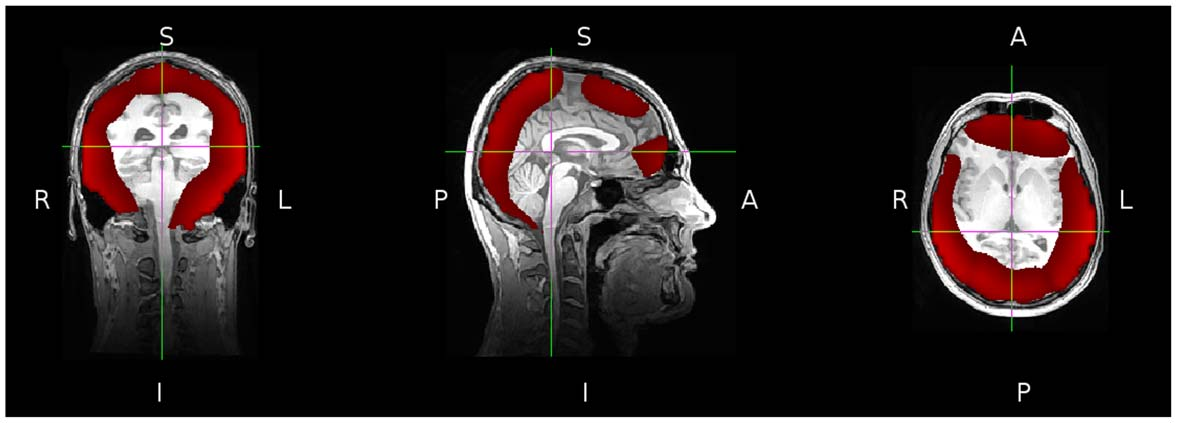
Volumetric image showing magnitudes of the gains of sources inaccurately reconstructed as being in the 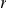 direction when the embedded source was in the tangential plane (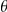 or 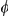 direction) when using three 1-dimensional filters. The image has been thresholded to show the magnitude of the gain being greater or equal to 1.0.

The 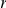 reconstructions show both a high gain and correlation when the source was embedded in the 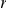 direction, as previously discussed. When the signal was seeded in a non-radial direction, the reconstructions in the 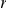 direction show a combination of high gains and high correlations. 32.56% of the locations were found to have a gain magnitude greater than 1 in the 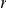 direction when no source was seeded in this orientation (range −4.56 to 5.49). 40.07% of the volume was found to have a correlation greater than 0.7. Figure 4 shows a volumetric image of the magnitude of the gain at each location in the 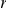 source reconstruction when a signal was seeded in one of the two tangential directions. The image has been thresholded to only show locations where the magnitude of the gain was above 1. As the overlay in red shows, there are large parts of the volume where the reconstruction in the 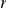 direction has a gain magnitude greater than 1 even though no source was seeded in this orientation. These locations are predominantly superficial.

### Reconstruction using a three-dimensional spatial filter

**Figure 5:**
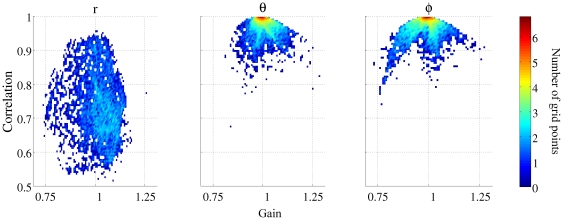
Two dimensional histogram of gain and correlation of the reconstruction compared with the embedded signal at each location in the brain volume for one 3-dimensional filter. The left, middle and right columns show the results for sources both embedded and reconstructed in the 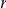, 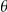 and 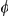 directions respectively.

Figure 5 shows the gain and correlation for the time series reconstructions in 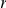, 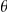 and 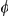 when using a three-dimensional implementation of a vectorised beamformer, i.e. the construction of the filter constrains the pass characteristics of the off-directions.

Figure 5 is analogous to figure 1 and again shows that reconstructions in the tangential directions have a gain magnitude and correlation both around 1 (mean = 0.95, std = 0.06). As in the case of the one-dimensional spatial filter, the reconstructions in the 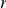 direction again show lower correlations (mean = 0.73, std = 0.10). The percentage of locations with a correlation above 0.7 are now 60.41%, 99.08% and 99.05% in the 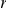, 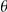 and 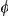 directions respectively. The results shown in figure 5 suggest that when the time series reconstruction is performed in the correct orientation, both the one- and three-dimensional spatial filter implementations perform comparably, although in the 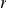 direction, the three 1-d filter implementation performs slightly better than the single 3-d filter.

**Figure 6:**
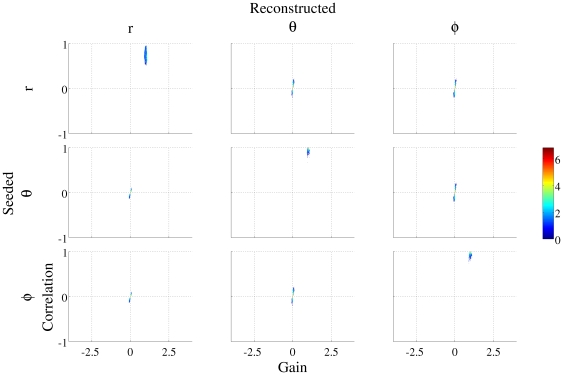
Two dimensional histograms of gain and correlation of the reconstruction compared with the embedded signal at each location in the brain volume for one 3-d filter. The upper, middle and lower rows represent sources *seeded* in 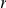, 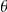 and 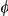 respectively; whilst the left, middle and right hand columns show *reconstructions* in 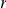, 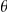 and 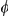. The diagonal of these figures shows the same data as that in Figure 5.

Figure 6 shows that the results in the off-directions are both quantitatively and qualitatively different to the one-dimensional implementation results shown in figure 3. The reconstruction in the two directions orthogonal to the embedded source orientation now show gains and correlations closely centered around zero. Across all the grid points in which a time series reconstruction was performed in the non-embedded direction (six experiments of 14793 grid points; giving 88,758 reconstructions) the range of the gains was −0.22 to 0.23 and the range of correlations observed was −0.11 to 0.16. This suggests that the “leaking” of the signal into other orientations which was seen in the one-dimensional filter implementation is not present when one 3-dimensional filter is used.

### Experimental source localisation

**Figure 7:**
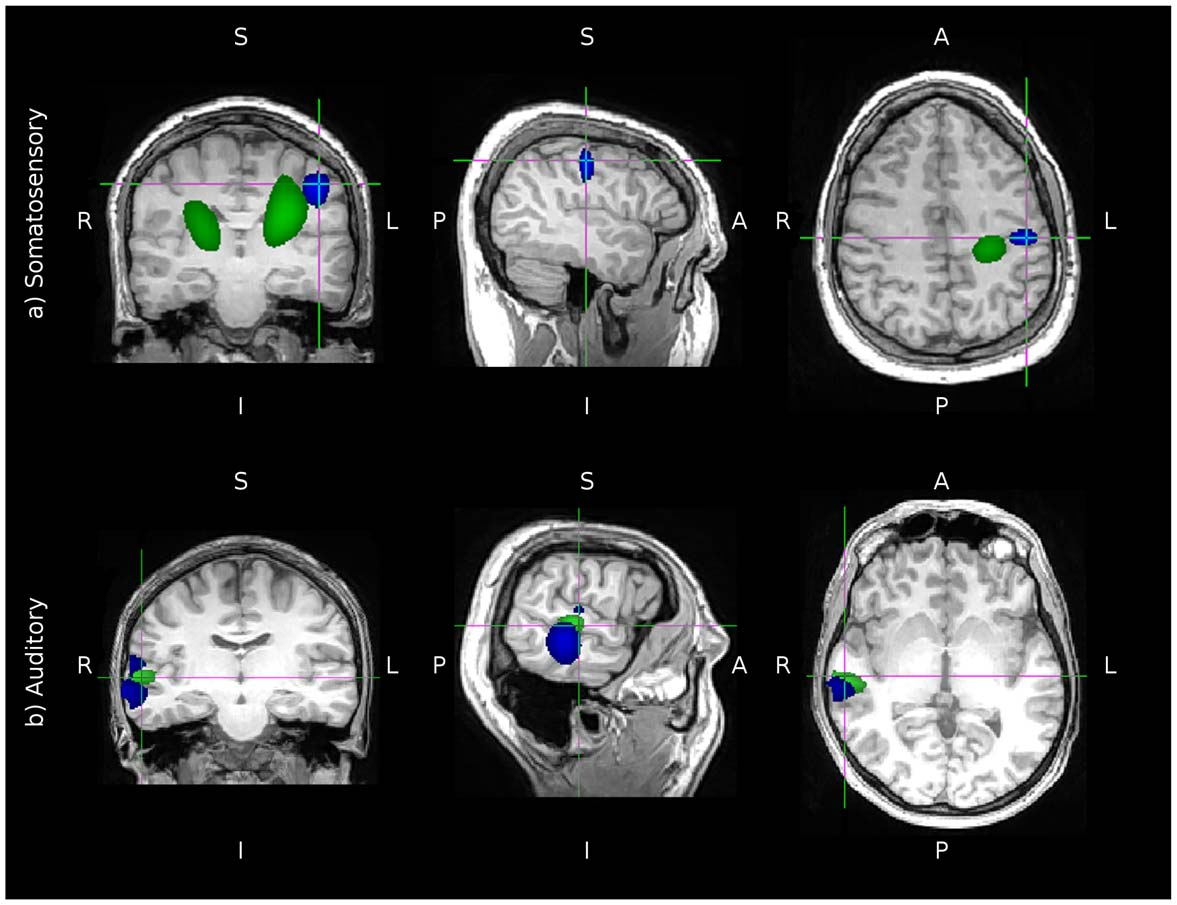
Comparison between three 1-d filters and one 3-d filter in experimental data. The blue overlay shows the combined output of three 1-d filters whilst the green overlay shows the combined output of one 3-d filter. Images are shown in radiological convention. Subfigure (a) shows 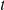-maps between active and passive normalised power measures in a somatosensory stimulation experiment. Arbitrary thresholds were applied to only show the peak in the map. In blue are regions with a t-value between 10.5 and a maximum of 12 and in green between 3 and a maximum of 4.5. Subfigure (b) shows 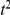 maps evaluating the 4 Hz Fourier component in an auditory steady state following response. Arbitrary thresholds were applied to only show the peak in the map. In blue are regions with a t-squared value between 550 and a maximum of 900 and in green between 350 and a maximum of 450.

The three 1-dimensional and one 3-dimensional spatial filter implementations were also used to localise responses from human sensory experiments. The first data involved a somatosensory stimulation experiment. The analysis windows used to localise the response were a pre-stimulus baseline period of −300 to −50 ms pre-stimulus-onset and an “active” period of 50–300 ms post-stimulus-onset. These time intervals were used to estimate the source power throughout the volume and a 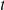-test was subsequently performed on these power maps. Although a 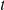-test was performed, only the peak in the map was considered for analysis. No statistical thresholds were applied. Figure 7a shows in blue the peak in the 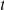-map generated using the three 1-dimensional filters approach. The overlay in green shows the 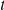-map for the exact same analysis conducted using the one 3-dimensional filter approach. The slice selections are centred over the left primary somatosensory cortex and whilst the peak for the 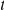-map created using three 1-dimensional filters is found in this region, the 3-dimensional filter does not localise the response to primary somatosensory cortex.

The second experimental dataset consisted of the response to an auditory steady state stimulus. At each point on a 5 mm grid placed throughout the volume, a 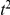 statistic was calculated using the magnitude and phase of the 4 Hz Fourier component across all 239, one-second epochs. This analysis was performed using both the three 1-dimensional and one 3-dimensional spatial filters. The peaks in the 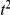 maps are shown plotted in blue (three 1-d case) and green (one 3-d case) in figure 7b for a single individual. The peak in the 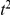-map for the summed output of three 1-dimensional filters shows a primary peak in the inferior portion of the temporal lobe. This is accompanied by a secondary peak in the superior plane of the temporal lobe. There is a decrease in 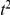 values in-between these two peaks. It is at this location that the maxima in the 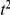-map for the one, 3-dimensional filter is seen. The exact same analysis path was followed to generate both maps. For each component of the vectorised beamformer outputs, a 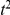 value was calculated across epochs and these were then summed at each point to give an estimate of the total 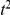 value for the Fourier component of interest at each point. The only difference in the analyses is the initial construction of the spatial filters, whether the inverse problem was posed as an integrated 3-dimensional problem or three independent 1-dimensional problems.

### Simulated source localisation

It is, of course, informative and essential to investigate the extent to which the different filter implementations affect the volumetric images produced when analysing real data. However, the difficulty with this approach is that the “true” location of activity is unknown and can at best only be estimated. In order to further compare the ability of the two filter designs to localise activity through a comparison based on power measures, a series of simulation experiments were performed. This was a replication of an experiment described in [9] in which a superficial dipole was moved from a medial-superior location to a lateral-inferior position in 5 steps. Each of the 5 dipoles was seeded independently in separate analyses, so there was only ever one source present. The dipole was oriented in the *y* direction (left-right axis). Each of these analyses was repeated 30 times with the only difference being the background oscillatory activity in which the signal was embedded. This allows an estimate of the variability of the metric to be obtained. The signal embedded and the method were identical to those described in the methods, with the exception that only one location was seeded and an NAI was calculated for this active and a passive window and a t-test performed between the two.

**Figure 8:**
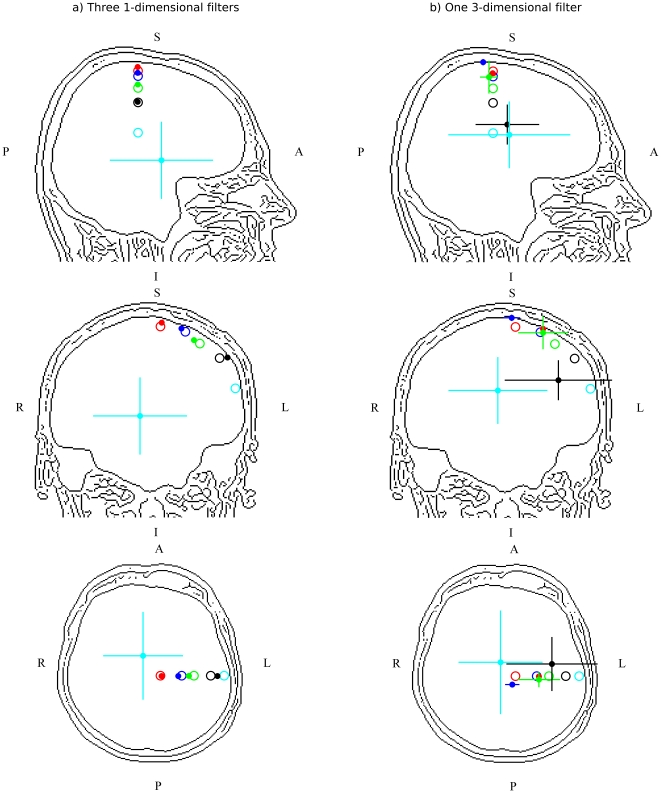
Comparison of the two filter implementations and their ability to localise a single embedded source. The open circles represent the seeded location, the filled circles represent the average localisation across 30 experiments (with 1 standard deviation shown as cross-hairs). Subfigure (a) shows three 1-dimensional filters. Subfigure (b) shows one 3-dimensional filter.

Figure 8 shows the location of the 5 seeded dipoles as open circles and the localisation from the t-maps are shown as a solid circle. This localisation was obtained by averaging the location from the 30 independent experiments. Cross-hairs are used to show one standard deviation of the localisation across the 30 different noise permutations. Figure 8a shows the localisations obtained using three 1-dimensional filters. The four most superior locations are localised accurately to the closest grid point (as the dipoles were not seeded on the actual grid used for the analysis). The lack of cross-hairs for these experiments indicate that the localisation was consistent. The most lateral and inferior dipole was localised both inaccurately and inconsistently, and therefore accurately replicate the findings from the initial experiment described by [9].

Figure 8b shows the same analysis, of the same experimental conditions when using one 3-dimensional filter. The four most superior locations are now poorly localised and the cross-hairs present on the third and fourth locations (green and black) confirm that there is more variability across repeated experimental runs. The results suggest that when localising a response on a power-based metric, such as the NAI, the implementation described by Huang is potentially a more accurate, and more reliable method with which to accurately identify the underlying neuronal source.

## Discussion

In this paper we investigated one- and three-dimensional implementations of spatial filters applied to synthetic MEG data. The one-dimensional filter implementation constructed three independent filters in orthogonal directions at each location. The three-dimensional filter implementation constructed the filters as a three-dimensional system and therefore included the constraint that the pass characteristics of the off-direction terms must have zero gain.

The results presented confirm that if a time series reconstruction is performed in the same orientation as the source was placed, there is little difference between the two implementations of the beamformer. In the non-radial directions the reconstructed time series at each point showed a gain and correlation both around 1. In the predominantly radial direction, although the correlations were lower than in the tangential directions, 60.41% remained above 0.7 at all grid locations. It was shown that in general, deeper source locations showed lower levels of correlation. The slight improvement in the three 1-d filters is not surprising given that the additional constraints placed on the 3-d filter reduce the degrees of freedom the filter has to minimise power from the rest of the volume. This will result in noisier reconstructions, although the results presented here suggest that the difference is small and that both implementations can successfully reconstruct predominantly radial sources. This confirms that a vectorised beamformer is able to accurately reconstruct activity from any location within the volume, regardless of orientation. However, deeper sources were reconstructed with less accuracy when sources were oriented radially. This is in concordance with work done using Equivalent Current Dipoles to investigate sensitivity in gradiometer-based systems which suggested that depth rather than orientation was the determining factor of the sensitivity of the model [17].

In the one-dimensional implementation, the large gain magnitudes and correlations observed in the off-directions may potentially pose a problem. The large gain magnitudes seen in the 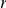 direction in the absence of any signal seeded in this orientation could cause a problem for any localisation metric which relies on power. A number of techniques restrict themself to the tangential plane, most notably SAM [3]. It is often assumed in the literature that this is due to an inherent problem with radial sources, however the current work suggests that this is not a generic issue but may be the result of specific implementation choices with regard to spatial filter construction and the use of a single sphere model. However, a number of recent approaches have used a spatial filter to perform source localisation focusing on stimulus characteristics other than source power (e.g. [8], [9]). For these metrics, the occurrence of high correlations in the absence of any source seeded in the orientation regardless of the gain of the source could also lead to errors in source localisation.

The results presented highlight a number of issues to be aware of when using outputs from any spatial filter construction. This is particularly important when using a scalar beamformer which adaptively selects the orientation for subsequent analysis. If a signal is oriented predominantly radially, it is possible to accurately reconstruct both the shape and the magnitude of this signal using a spatial filter. This was demonstrated using both a one- and three-dimensional spatial filter. Therefore, if an external stimulus in an experiment were to elicit a response that is predominantly radial, subsequently constraining the analysis to the tangential plane would lead to a reduction in accuracy of the analysis. The three-dimensional spatial filter described by Van Veen et al is able to provide an estimate of phase locked source activity in three orthogonal directions without being biased by the predominantly radial sources. If one decides it is advantageous to constrain the analysis to the tangential plane, the orientation must still be determined and the effects of different filter construction still apply. There are several methods of choosing the optimal orientation within the tangential plane (e.g. [18]) and the effects described in this work could bias the direction that is found, depending on what constitutes “optimal” for a given method.

The time series reconstruction obtained when using a three-dimensional spatial filter produced low gains and low correlations in the directions where no source was placed. This method therefore yields a more accurate estimate of the three-dimensional neural time course. Virtual electrode reconstruction and source localisation are however, not the same problem. Huang et al [13] modified the spatial filtering approach in order to reduce the susceptibility to single orientation noise dominance in source localisation. The somatosensory results presented show that power-based localisation is closer to somatosensory cortex when using the modification as proposed by Huang et al based on *a priori* knowledge of the anatomical location of primary somatosensory cortices. In addition, preliminary simulation results support the claim that the modification proposed by Huang et al results in a power localisation metric which is both more accurate and less susceptible to changing background activity than the original three-dimensional filter described by van Veen et al. Conversely when performing source localisation on metrics which are more reliant on accurate source reconstruction, such as examining the phase and magnitude of a response frequency as shown in the the auditory data presented, our results suggest that a three-dimensional spatial filter may be advantageous. Therefore it may be that the optimum method with which to construct the spatial filters depends on the type of response being investigated. If a power-based metric, such as the commonly implemented pseudo-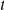 evaluation of pseudo-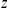 scores is used then it clearly is important to noise correct on a per-orientation basis, although the modification made by Huang et al is not without its drawbacks. It may be possible to perform per-orientation noise normalisation whilst maintaining an integrated, 3-dimensional approach to the construction of the spatial filter. The results presented highlight some of the problems inherent in using power as a metric for localisition. Power must be normalised, but the current methods do so at the cost of accurate source reconstruction and can lead to large errors in the estimation of non-tangential sources. In this regard, metrics which focus on characteristics of source reconstructions which do not require noise normalisation have clear advantages over standard power based localisations.

The work presented in this paper utilised both a one- and three-dimensional spatial filter. This is distinct from the implementation of a scalar or vector beamformer. Scalar and vector beamformers are commonly taken to describe the number of components present in a source estimation or reconstruction. The two analyses presented in this paper would be both be described as a vectorised beamformer as each one produced three orthogonal estimates of source activity at each grid point. The difference between the two analyses is whether the filter is constructed as an integrated three-dimensional system or as three independent one-dimensional problems. A scalar beamformer can be calculated using either a one- or three-dimensional spatial filter. Once the orientation of the scalar beamformer is determined, the activity in this direction can be estimated by constraining the activity in the off-terms or by leaving them to vary freely. Therefore if a scalar beamformer is used to investigate a given neural response, care must be taken in how the filter is setup and whether this source estimate is created from a three- or a one-dimensional system.

Although the differences between the two spatial filter implementations are described in the literature, the consequences of these differences have not been fully explored and have received much less attention than whether a particular beamformer output is considered scalar or vectorised. The majority of papers using spatial filtering do not explicitly state how the analysis has been performed or how the filters were constructed. Often, mathematical notations are unclear and poorly stated. It is clear that there are differences between the two implementations, both for virtual electrode reconstruction and source localisation and therefore we recommend that authors are more explicit in their descriptions of the spatial filtering techniques used. This will allow greater replicability of results and methods.

## Methods

### Simulated signal

For the simulation experiments described, a system of coupled oscillators was used to provide a source. Coupled oscillators have been used successfully to model neural sources (see for example [19]). In this paper, the coupled oscillators used are described by the following differential equations, where 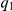 and 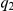 are the states of the system and 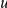 is the input [20]:
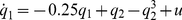
(11)

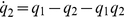
(12)The input used when generating the two states was broadband noise low-pass filtered at 100 Hz. This resulted in oscillatory output time series, with the majority of the power in the 0–40 Hz range, and an lower magnitude high frequency tail. 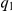 was taken as the source for all epochs of all experiments described. The signal strength used for the simulations were set to yield a phase-locked response on the sensor array in the same range as those found in real experimental data [9].

### Intrinsic Brain Activity

All recordings were made at York Neuroimaging Centre on a 248 channel whole-head magnetometer system (4D NeuroImaging) with a sampling rate of 678.17 Hz and a 200 Hz bandwidth. Intrinsic brain activity was collected from a healthy 22 year-old male with no known brain pathologies. The recording was made with the subject's eyes open and a black fixation cross presented on a white screen. The acquisition lasted for 10 minutes and the signal was divided into epochs of 1.4 s duration (961 data points). Two EOG channels were used to reject epochs containing large signals related to eye movements and from the remaining data, the first 100 clean epochs were used for the simulation experiments. Ethical permission was obtained from the Research Governance Committee of York NeuroImaging Centre, University of York and written, informed consent was obtained from the subject prior to scanning.

### Simulation Paradigm

A 5 mm grid was placed throughout the cortical volume of the participant, which yielded 14793 points. Each of these grid points was analysed independently in a single analysis, with the same analysis being carried out with the signal embedded in each of the three directions, 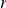, 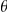 and 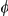. The same 100 epochs of intrinsic brain activity were used for each point and orientation. The analysis performed was as follows for each of three seeding directions 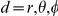;

1. The simulated signal was embedded in each of 100 epochs at a point within the brain, 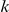, and in a specific orientation, 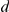.
2. A time series reconstruction was performed at the point of interest in three orthogonal directions and the phase-locked signal was obtained by averaging this reconstruction across epochs.
3. A linear regression was then performed on the embedded signal and each of the source reconstructions performed in 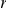, 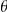 and 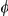, i.e. the direction of embedding and the two orthogonal directions.
4. This process was repeated at each of the 14793 grid points.

The correlation of the two time series and the slope of the regression were used to evaluate the accuracy of the source reconstruction. The optimum outcome for the spatial filter is to reconstruct a signal in the correct orientation with a correlation and a gain of one, whilst also showing low correlation and a gain around zero for the reconstructions in the off-directions, i.e. the two orthogonal orientations in which the source was not embedded.

### Experimental data

In addition to the simulation experiments performed, two experimental datasets were analysed. Both experiments were recorded using the 4-D Neuroimaging MEG scanner previously described and were also recorded at a sample rate of 678.17 Hz with a bandwidth of 200 Hz.

The first was a somatosensory experiment, full details of which can be found in Hymers et al [9]. A plastic diaphragm was used to stimulate the right index finger. The duration of stimulation was around 200–250 ms and 150 epochs were presented with a 1.5 s inter-stimulus-interval. The analysis was conducted by performing a t-test on NAI maps calculated from an active and passive period. The active window was defined as 50 ms post-trigger to 300 ms post-trigger and the passive window was 300 ms pre-trigger to 50 ms pre-trigger. The beamformer weights were calculated separately for active and passive windows.

The second dataset contained a measure of the auditory steady-state response. The experiment consisted of diotically presented 500 Hz carrier tones, amplitude modulated at 4 Hz. Full details of the experimental procedures can be found in [21], but in summary, an amplitude modulated sound was presented for 239 seconds and this was segmented into one-second epochs. The 4 Hz Fourier component was calculated for each epoch and these values were subjected to a t-squared test in order to evaluate the magnitude and phase of the frequency component of interest.
